# Auto-induction expression of human consensus interferon-alpha in *Escherichia coli*

**DOI:** 10.1186/s12896-015-0128-x

**Published:** 2015-03-06

**Authors:** Nawal Abd EL-Baky, Mustafa H Linjawi, Elrashdy M Redwan

**Affiliations:** Therapeutic and Protective Proteins Laboratory, Protein Research Department, Genetic Engineering and Biotechnology Research Institute, City for Scientific Research and Technology Applications, New Borg EL-Arab, 21934 Alexandria, Egypt; College of Applied Medical Sciences, King Abdulaziz University, P.O. Box 80203, Jeddah, 21589 Saudi Arabia; Biological Sciences Department, Faculty of Science, King Abdulaziz University, P.O. Box 80203, Jeddah, 21589 Saudi Arabia

**Keywords:** Auto-induction, Human consensus interferon-alpha, IPTG-inducible expression system, T7 expression system

## Abstract

**Background:**

Isopropyl-β-D-1-thiolgalactopyranoside (IPTG)-inducible expression of recombinant proteins in *E. coli* is commonly used and effective. Nevertheless, unintended induction was encountered as a problem when using these bacterial expression systems, generating cultures that give reduced or variable protein yields. Auto-induction allows for production of much higher target protein yield and cell mass than conventional procedures using induction with IPTG without monitoring cell growth then adding IPTG at the appropriate cell density. This method involves special media recipes that promote growth to high density and automatically induce expression of target protein from T7 promoter. Consensus interferon is a synthetic artificially engineered interferon having an amino acid sequence that is a rough average of the sequences of all natural human alpha interferon subtypes and has greater potency than other interferons even the pegylated versions. The purpose of this study was high-level expression of human consensus interferon-alpha (cIFN-α) in *E. coli* using an auto-induction protocol. The cIFN-α gene was cloned into pET101/D-TOPO expression vector under the T7 promoter transcriptional regulation. Expression was optimized with respect to temperature and length of incubation in shake flask cultures. The antiviral potency and anticancer activity of cIFN-α were evaluated in comparison to IFN-α2a.

**Results:**

The expressed cIFN-α protein in auto-induction T7 system was found mostly in soluble fraction of the cell lysate (about 70% of yield in total cell lysate) after lowering incubation temperature to 25°C or 30°C. Protein expression was maximal after 24 h incubation at 25°C or 30°C. After purification via single-step chromatography using DEAE-Sepharose, the yield was 270 mg/L in shake flask *E. coli* cultures which is much higher than IPTG-inducible T7 expression system and other systems according to available data. The synthesized cIFN-α was biologically active as confirmed by its anticancer and antiviral effects and was significantly more potent than IFN-α2a.

**Conclusions:**

The auto-induction process was reliable and convenient for production of cIFN-α protein in *E. coli*, and can be adapted for large-scale therapeutic protein production.

## Background

Interferons (IFNs) are cytokines secreted by vertebrates’ cells when stimulated by viruses and several other agents [1,2]. This family of glycoproteins exhibits antiviral, antiproliferative and immunomodulation functions [3]. Based on their receptor specificity, structure and functional properties, IFNs are divided into 3 major groups (type I (IFN-α and IFN-β), type II (IFN-γ) and type III (IFN-λ)) [4]. Consensus interferon is a synthetic type of IFN-α containing 166 amino acids that was engineered to comprise the most frequently observed amino acids among all natural non-allelic IFN-α subtypes in humans [5] to form a consensus molecule. *In vitro* studies have shown that consensus interferon yields more potent antiviral and antiproliferative effects than other standard interferons (IFN-α2a, IFN-α2b, and pegylated versions) [6]. This artificial interferon was used in combination with ribavirin to treat non-responder population or relapsers to a previous interferon regime [7,8].

There have been many efforts to express the artificial gene of consensus interferon in *E. coli* [9-12]. Fieschko and Ritch [10] developed a semisynthetic medium for the production of human consensus interferon-α (IFN-alfacon-1) via tightly regulated expression system and a controlled feeding schedule. They obtained yields up to 7.6 × 10^12^ U per liter of fermentation broth, however this result was not reproducible. Curless *et al*. [12] designed cyclic, two-stage fed-batch cultivation schemes for the expression of human consensus IFN-α under the λP_L_ promoter transcriptional control. They achieved a yield of 5.5 g/L interferon at a cell density of 68 g of dry cell weight (DCW)/L but maintaining two reactors may have restricted the use of this design. We have previously reported heat-inducible expression of cIFN-α protein in *E. coli* with a λP_L_ promoter system [13]. Most of the expressed protein aggregated into inclusion bodies, therefore needed *in vitro* refolding. After refolding and purification, the yield was 70 mg/L in shake flask cultures.

The T7 system comprising the T7 promoter and phage T7 RNA polymerase is one of the most widely used expression systems [14]. In host *E. coli* strains such as BL21 (DE3), T7 RNA polymerase is expressed under the control of the IPTG-inducible *lac*UV5 promoter. Expression of the target protein from cloned gene is controlled by the T7 promoter. This promoter is recognized specifically by T7 RNA polymerase. After sufficient amount of T7 RNA polymerase is produced, it binds to the T7 promoter and transcribes the gene of interest. Various recombinant proteins were overexpressed in *E. coli* using this system. A problem in using this expression system is that there is always some basal expression of T7 RNA polymerase from the *lac*UV5 promoter even in the absence of inducer [14]. If the target protein is toxic to the *E. coli* host, basal expression of the target gene possibly will result in plasmid instability and/or cell death. To reduce basal expression, a *lac* operator sequence (a binding site for *lac* repressor) is placed downstream of the T7 promoter, constructing a T7*lac* promoter [15]. The *lac* repressor binds to the *lac* operator and functions to further repress T7 RNA polymerase-induced basal transcription of the target gene in BL21 (DE3) cells. In addition, IPTG induction frequently leads to formation of inclusion bodies [16].

Recently, Studier [17] developed an auto-induction process for production of recombinant proteins under the control of the T7 promoter in *E. coli*. Auto-inducing media are made from a well-balanced combination of different carbon sources and other essential nutrients, allowing cultures to grow to high cell densities, and support high-level expression of the target protein. These media contain glucose, which promotes fast cellular growth in the early stages of the culture, while also preventing uptake and metabolism of inducing sugar. Lactose is also present in these media, and is consumed after glucose exhaustion, inducing recombinant protein expression. To achieve sustained growth during the induction phase, glycerol is provided in auto-inducing media together with lactose. Formulation of auto-inducing media achieved higher recombinant protein yields than those using IPTG as inducer [17]. The aim of this work was expression of cIFN-α protein in *E. coli* by auto-induction procedure in shake flasks for the first time, purification of recombinant cIFN-α and determination of its antiproliferative and antiviral activities.

## Methods

### Cloning of the cIFN-α gene into pET101/D-TOPO expression vector

The coding sequence of the synthetic cIFN-α gene was assembled and amplified by two-step PCR-based gene synthesis method using long (45–50 nucleotides) overlapped primers and GC-Rich PCR system (Roche Molecular Biochemicals, Mannheim, Germany) as described previously [13,18]. The blunt-ended PCR product of cIFN-α gene was cloned into the pET101/D-TOPO vector (Invitrogen, Carlsbad, USA) as recommended by the manufacturer to yield pET-cIFNα expression construct. To allow pET directional TOPO cloning, the forward PCR primer used in amplification of the cIFN-α gene was designed to contain the sequence, CACC, at the 5′ end of the primer directly precedes an initiation ATG codon. The 4 nucleotides, CACC, base pair with the overhang sequence, GTGG, in pET-TOPO vector. A stop codon (TAA) was included in the reverse primer. The resulting pET-cIFNα plasmid was transformed into BL21-CodonPlus (DE3) chemically competent *E. coli* (Stratagene, Heidelberg, Germany) for protein expression regulated by the T7 promoter. Then the cells were plated onto Luria Bertani (LB) agar plates containing ampicillin (100 μg/ml). Plasmid was purified using Miniprep kit (Qiagen, Hilden, Germany) from overnight culture of picked single colonies then *Xba*I*/Sac*I enzymes digested. Correct clones were subjected to nucleotide sequencing.

### Auto-induction for consensus interferon production in *E. coli*

The Overnight Express Autoinduction System 1 (Novagen, Darmstadt, Germany) was employed for high-level cIFN-α protein expression by auto-induction from the constructed pET-cIFNα plasmid. The Overnight Express Autoinduction System 1 medium was prepared aseptically by adding 20 ml OnEx solution 1 (induction solution), 50 ml OnEx solution 2 (buffering solution), and 1 ml OnEx solution 3 (magnesium solution) to 929 ml sterile LB broth. A single clone was cultured in the Overnight Express Autoinduction System medium supplemented with 100 μg/ml ampicillin in a shaker incubator at 30°C and 300 rpm. After overnight or 24 h incubation, cells were harvested by centrifugation at 4000 rpm for 15 min at 4°C. Each cell pellet collected from 1 ml of the bacterial culture was resuspended in 100 μl of 5× electrophoresis sample buffer, boiled for 10 min, and then loaded onto sodium dodecyl sulfate polyacrylamide gel electrophoresis (SDS-PAGE). Auto-induced cultures were grown and induced at different incubation temperatures (25, 30, and 37°C) for 24 h, then soluble cell lysates of auto-induced cells were analyzed by SDS-PAGE.

Two immunoassays were performed to confirm the identity of the expressed protein in auto-induction T7 system; enzyme-linked immunosorbent assay (ELISA) and western blot. ELISA was performed as previously described [13,19]. Briefly, ELISA microtiter plate (Costar, Cambridge, USA) was coated with 50 μl of total cell lysate at a concentration of 5 μg/ml in carbonate buffer solution (pH 9.6) for 24 h at 4°C. After washing 5 times with 0.12 M NaCl, 0.04 M sodium phosphate, pH 7.2 buffer (PBS), the plate was blocked by adding 100 μl of blocking buffer (2% w/v gelatin in PBS) for 1 h at 37°C. Then the plate was washed 5 times with PBS and 50 μl of monoclonal mouse anti-human IFN-α antibody (PBL InterferonSource, Piscataway, USA) diluted 1:2000 was added to each well. After 1 h of incubation at 37°C, the plate was washed 5 times with PBS, and 50 μl of alkaline phosphatase-conjugated anti-mouse IgG (BIO-RAD, Alfred Nobel, Hercules, USA) diluted 1:1000 was added, followed by an incubation of 1 h at 37°C. After washing five times, *p*-Nitrophenyl phosphate (*p*-NPP) was added for color development and optical density was measured at 405 nm using an ELISA microtiter plate reader (MicroPlate Reader, BIO-RAD, USA). For western blotting, the protein bands were separated on 12% SDS-PAGE and transferred onto a nitrocellulose membrane in a buffer of pH 8.4 containing 1.93 g/L Tris base and 9 g/L glycine. Transfer was done for 8 h at 30 Volt. After transfer, the membrane was blocked using 2% w/v bovine serum albumin (BSA) in PBS for 1 h. After a brief wash with PBS, the membrane was incubated with monoclonal anti-human IFN-α antibody diluted 1:2000 for 1 h at room temperature with gentle shaking. The membrane was washed again three times with PBS for 10 min each, and then incubated with anti-mouse IgG-peroxidase conjugate (BIO-RAD, USA) diluted 1:1000 at room temperature for 1 h with gentle shaking. Washing was done again with the same procedure described above. Protein bands were developed with 3,3 Diaminobenzidine (DAB) [20].

### Optimization of auto-induction expression

To grow the cells to stationary phase (OD_600_ = 6–7) and optimize the yield of cIFN-α production in auto-induction system, cultures were incubated at different temperatures (25, 30, and 37°C) and samples were taken at different time points (8, 10, 12, and 24 h). Yields of cIFN-α (volumetric yields (g/L)) were calculated.

### Purification

The supernatant and the pellet (inclusion bodies) of the cell lysate were prepared from 1 liter of optimized auto-induced shake flask culture and then subjected to SDS-PAGE analysis as previously described [21]. The cell lysate supernatant was loaded at 1 ml/min onto a DEAE-Sepharose CL-6B anion-exchange column (Pharmacia Biotech, Uppsala, Sweden) previously equilibrated with 20 mM Tris–HCl, pH 8. The column was washed with ten bed volumes of the same buffer (flow rate 2 ml/min). The bound proteins were eluted with 100 ml linear gradient of 0–1 M NaCl generated using AKTAprime plus FPLC protein separator system (GE Healthcare Life Sciences Products, Cardiff, UK) at a flow rate of 1 ml/min. Twenty fractions of 5 ml each were collected. The eluted protein in the fractions was monitored by an online UV detector. Purified cIFN-α protein was analyzed by SDS-PAGE and ELISA. Purified cIFN-α protein content was determined by Lowry method [22]. The endotoxin content was checked [23] to avoid its mitogenic effects on the cell culture system. All cIFN-α preparations used were free of endotoxin (data not shown).

### Cell culture

Hepatoma cell line HepG2 HB-8065 (ATCC, USA) was cultured in RPMI-1640 medium (Lonza, Verviers, Belgium) supplemented with 2% v/v L-glutamine, 10% v/v fetal bovine serum (FBS), and 1% v/v penicillin-streptomycin. The cultures were maintained at 37°C in a humidified atmosphere with 5% CO_2_; 95% air in 25 cm^2^ flasks (Greiner, Frickenhausen, Germany). For subculturing, culture medium was discarded, and then the adherent cells were detached from the surface of the flask using 1 ml of Trypsin-EDTA solution (0.25% w/v Trypsin- 0.53 mM EDTA) for 5 to 15 min at 37°C. The action of Trypsin was stopped by the addition of 3 ml RPMI-1640 medium. The cells were scraped and collected in a 15 ml conical tube, then washed twice by RPMI-1640 supplemented medium, and centrifuged at 1200 rpm for 5 min at 4°C after each wash. The pellet was resuspended in 3 ml RPMI-1640 medium, and then appropriate aliquots of the cell suspension were added to new culture vessels.

### Anticancer activity assay

Recombinant cIFN-α expressed in auto-induction system was tested for anticancer activity against hepatoma cell line HepG2 and compared with commercial recombinant human IFN-α2a (Shenyang Pharmaceutical Co, Guoyaozhunzi, China) by using 3-(4, 5-dimethylthiazol-2-yl)-2, 5-diphenyltetrazolium bromide (MTT) rapid colorimetric assay as described by Mosmann [24] and El-Baky *et al*. [19]. Interferons were diluted with RPMI-1640 medium to desired concentrations (3, 3.33, 3.75, 4.28, 5, 6, 7.5, 10, 15 and 30 μg/ml). HepG2 cells were seeded in flat-bottomed 96-well cell culture plates at a density of 25×10^4^ cells/ml in final volume of 200 μl RPMI-1640 supplemented medium per well, and incubated for 24 h at 37°C in 5% CO_2_; 95% air. After 24 h, cells were treated with various concentrations of the tested interferons in 4 replicates. After further 24 h, the medium was discarded and cell monolayers were washed with sterile PBS three times. Twenty microliters of 5 mg/ml MTT solution (Sigma, St. Louis, USA) were added to each well and incubated at 37°C for 3 h. The formed insoluble purple formazan product was dissolved with 180 μl of dimethyl sulfoxide (DMSO). Optical density was measured at 560 nm, and then the percentage of cytotoxicity compared to the untreated cells was determined with the following equation:

$$ \%\mathrm{Cytotoxicity} = \frac{\mathrm{Absorbance}\ \mathrm{of}\ \mathrm{cells}\ \mathrm{with}\mathrm{out}\ \mathrm{treatment} - \mathrm{Absorbance}\ \mathrm{of}\ \mathrm{cells}\ \mathrm{with}\ \mathrm{treatment}}{\mathrm{Absorbance}\ \mathrm{of}\ \mathrm{cells}\ \mathrm{with}\mathrm{out}\ \mathrm{treatment}} $$

The plot of % cytotoxicity versus sample concentration was used to calculate the concentration lethal to 50% of the cells (LD_50_) value.

### Isolation of human blood lymphocytes and selectivity index calculation

The peripheral blood mononuclear cells (PBMCs) were isolated as previously described by Lohr *et al.* [25] and El-Fakharany *et al.* [26]. In brief, peripheral blood samples collected from a single healthy volunteer were diluted with 5 volumes of a freshly prepared red blood cell (RBC) lysis buffer of pH 8 containing 38.8 mM NH_4_Cl, 2.5 mM KHCO_3_, and 1 mM EDTA, incubated at room temperature for 10 min and centrifuged at 1500 rpm for 5 min. The nucleated cells were precipitated and washed with PBS. Proliferation of human lymphocytes in response to the tested interferons was assessed by MTT assay. The selectivity index (SI) value was calculated from the LD_50_ ratio in lymphocytes over HepG2 cells. The SI value indicates the selectivity of interferon samples to the HepG2 cell line [27].

### Antiviral assay

The antiviral activity of the tested interferons was determined using cytopathic effect (CPE) assay against herpes simplex virus 1 (HSV-1) as reported by Rashad *et al.* [28]. Vero cells grown to confluency in 96-well plates were infected with 100 μl of stock virus. After an adsorption period of 2 h at 37°C, virus was removed and the following concentrations of the tested interferons were added: 0.003, 0.03, 0.3, 3, and 30 μg/ml for recombinant cIFN-α and 0.1, 1, 10, 100, and 1000 IU/ml for commercial IFN-α2a, then maintenance Dulbecco’s modified Eagle medium (DMEM) with 2% v/v FBS was added (100 μl/well). The cultures were further incubated at 37°C for 24 h, until complete CPE was observed in the infected and untreated virus control. The determination of the anti-HSV-1 activity of the tested interferons was based on virus-induced cytopathogenicity of HSV-1 on Vero cells, measured at day 2 post virus infection by the MTT colorimetric method. An absorbance of formazan was detected at 560 nm. The results were expressed as the 50% effective concentration (EC_50_). The EC_50_ was defined as the interferon concentration required for protecting 50% of the virus-infected cells against viral cytopathogenicity. The concentrations of recombinant cIFN-α and commercial IFN-α2a which exhibited 50% cytotoxicity (LD_50_) were also determined. The therapeutic index was calculated by dividing LD_50_ on EC_50_.

### Statistical analysis

Differences between the variants were tested using Student’s *t*-test and McNemar’s test [29]. A *P*-value of <0.05 was considered statistically significant.

## Results

### Plasmid construction for auto-induction expression

**Figure 1:**
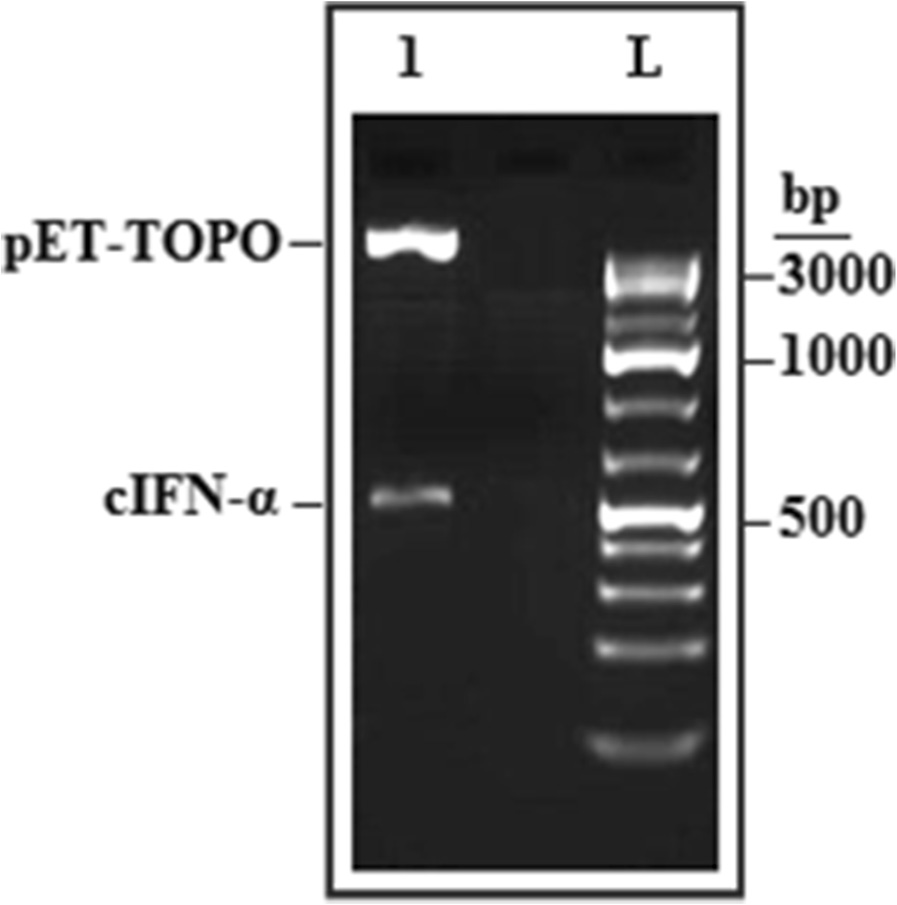
**A 2% agarose gel showing the result of restriction digestion of recombinant pET-cIFNα.** Recombinant pET-cIFNα was digested overnight at 37°C using *Xba*I and *Sac*I enzymes. Lane 1 shows the pET-TOPO vector fragment (5.73 kb) and the cIFN-α band (534 bp, with 26 bp added to the actual size of the gene from the vector cloning site and 10 bp introduced by gene-specific primers in the gene to enable pET directional TOPO cloning). L pointed GeneRuler 100 bp Plus DNA Ladder.

The blunt-ended PCR product of the synthetic gene coding for cIFN-α was directionally cloned into pET101/D-TOPO vector by adding four bases (CACC) to the forward primer. The overhang in the vector (GTGG) invades the 5′ end of the PCR product, anneals to the added bases, and stabilizes the PCR product in the correct orientation. The constructed pET-cIFNα plasmid was transformed into *E. coli* BL21 (DE3) for cIFN-α auto-induction expression under the control of the T7 promoter. One transformant harbored recombinant pET-cIFNα was analyzed by restriction digestion to confirm the presence of the insert and nucleotide sequencing. The sequencing data indicated that the transformant carried the correct construct (data not shown). As showed in Figure 1, the construct has the correct sized cIFN-α gene.

### Expression of consensus interferon by auto-induction

**Figure 2:**
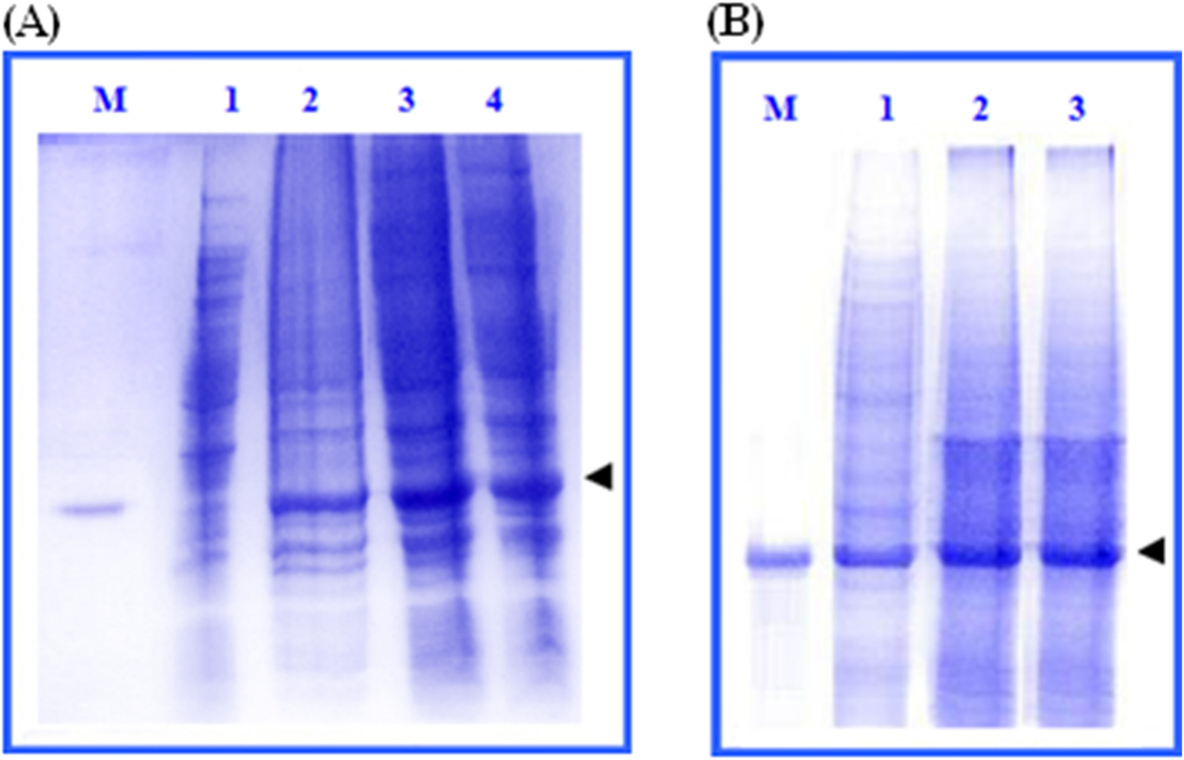
**SDS-PAGE analysis of auto-induction expression of cIFN-α. (A)** Auto-induction expression of cIFN-α at 30°C for 12 and 24 h. Lane M: commercial IFN-α2a as molecular weight marker of 19,2 kDa size. Lane 1: total proteins from uninduced cells. Lane 2: total proteins from IPTG-induced cells. Lane 3: total proteins from auto-induced cells after 24 h incubation at 30°C. Lane 4: total proteins from auto-induced cells after overnight incubation at 30°C. **(B)** Auto-induction expression of soluble cIFN-α. Lane M: commercial IFN-α2a as molecular weight marker of 19,2 kDa size. Lanes 1–3, soluble cell lysates of auto-induced cells after 24 h incubation at 37°C, 30°C, and 25°C, respectively. The arrow heads pointed the position of cIFN-α.

**Figure 3:**
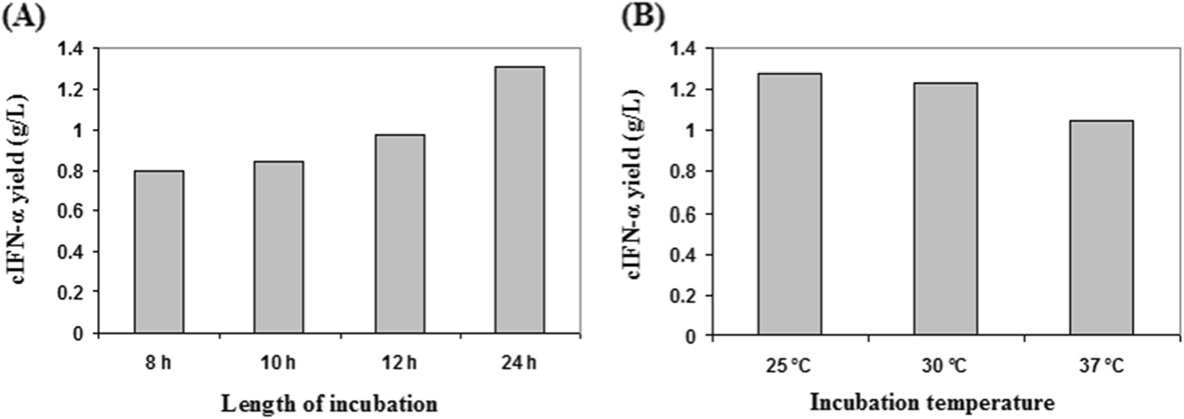
**Time and temperature optimization of cIFN-α auto-induction expression. (A)** Time optimization of cIFN-α auto-induction expression. Cultures were incubated for 8, 10, 12, and 24 h at 30°C. **(B)** Temperature optimization of cIFN-α auto-induction expression. Cultures were incubated at 25, 30, and 37°C for 24 h.

The recombinant human cIFN-α protein was overexpressed in auto-induced cultures using the pET system and lactose as inducer. After separation by 12% SDS-PAGE, total proteins obtained from auto-induced cells contained a thick protein band of about 19 kDa size, corresponding to the calculated size of cIFN-α protein (Figure 2A, lanes 3 and 4). SDS-PAGE analysis of crude proteins from auto-induced cultures or cultures induced by IPTG revealed that the auto-induced cultures yielded more cIFN-α protein than IPTG-induced cultures (Figure 2A, lanes 2–4). Figure 2A, lane 3 shows that maximum expression was reached by incubation of auto-induced cultures for 24 h at 30°C. Lowering incubation temperature to 25°C or 30°C improved the yield of soluble cIFN-α protein (Figure 2B). The effects of temperature and length of incubation on cIFN-α production level in auto-induction system are demonstrated in Figure 3.

**Figure 4:**
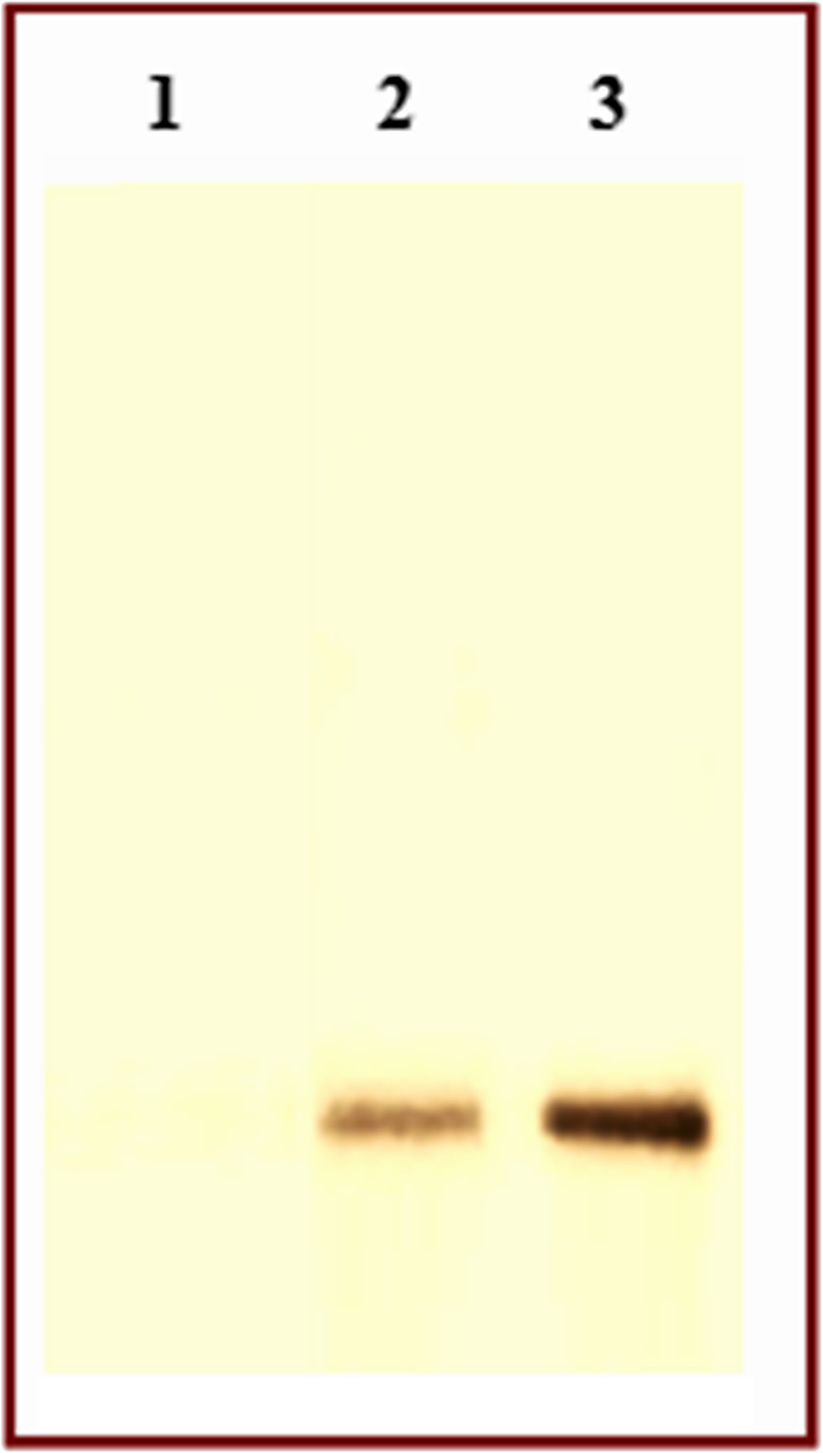
**Western blot analysis of expressed cIFN-α using the specific monoclonal anti-human IFN-α antibody.** Lane 1: total cell lysate of uninduced cells. Lane 2: commercial IFN-α2a as positive control and molecular weight marker of 19,2 kDa size. Lane 3: total cell lysate of auto-induced cells.

The identity of the overexpressed protein was verified by ELISA using monoclonal anti-human IFN-α antibody as presented in Table 1. The cIFN-α activity of cellular extract prepared from auto-induced *E. coli* BL21 (DE3) cells was presented as the mean ± standard deviation (SD) of three replicates. As demonstrated in Table 1, the mean absorbance values for cIFN-α in auto-induced cells and IFN-α2a were respectively 0.637 ± 0.022 and 0.934 ± 0.016, compared with the value of cIFN-α in cellular extract prepared from uninduced cells as negative control of 0.046 ± 0.008. A significant difference (*P* < 0.05) in ELISA signals was found between commercial IFN-α2a or recombinant cIFN-α in auto-induced cells and the negative control. ELISA results revealed that the auto-induced cultures yielded more cIFN-α protein than IPTG-induced cultures. Western blot analysis of expressed protein revealed that recombinant cIFN-α expressed in auto-induced cells was strongly and specifically recognized by monoclonal anti-human IFN-α antibody at the expected 19 kDa molecular weight (Figure 4).

**Table 1:** **Reactivity of monoclonal anti-human IFN-α antibody against recombinant cIFN-α expressed in** ***E. coli*** **BL21 (DE3) cells by auto-induction**

**Sample**	**OD at 405 nm (mean ± SD)**
Commercial IFN-α2a	0.934 ± 0.016
Recombinant cIFN-α (uninduced cells)	0.046 ± 0.008^a^
Recombinant cIFN-α (IPTG-induced cells)	0.447 ± 0.014^b^
Recombinant cIFN-α (auto-induced cells)	0.637 ± 0.022^a,b^

^a^Monoclonal anti-human IFN-α antibody reacted significantly (*P* < 0.05) with expressed cIFN-α protein in auto-induced cells.

^b^A significant difference (*P* < 0.05) in ELISA signals was found between recombinant cIFN-α in auto-induced cells and recombinant cIFN-α in IPTG-induced cells.

### Purification of expressed cIFN-α

**Figure 5:**
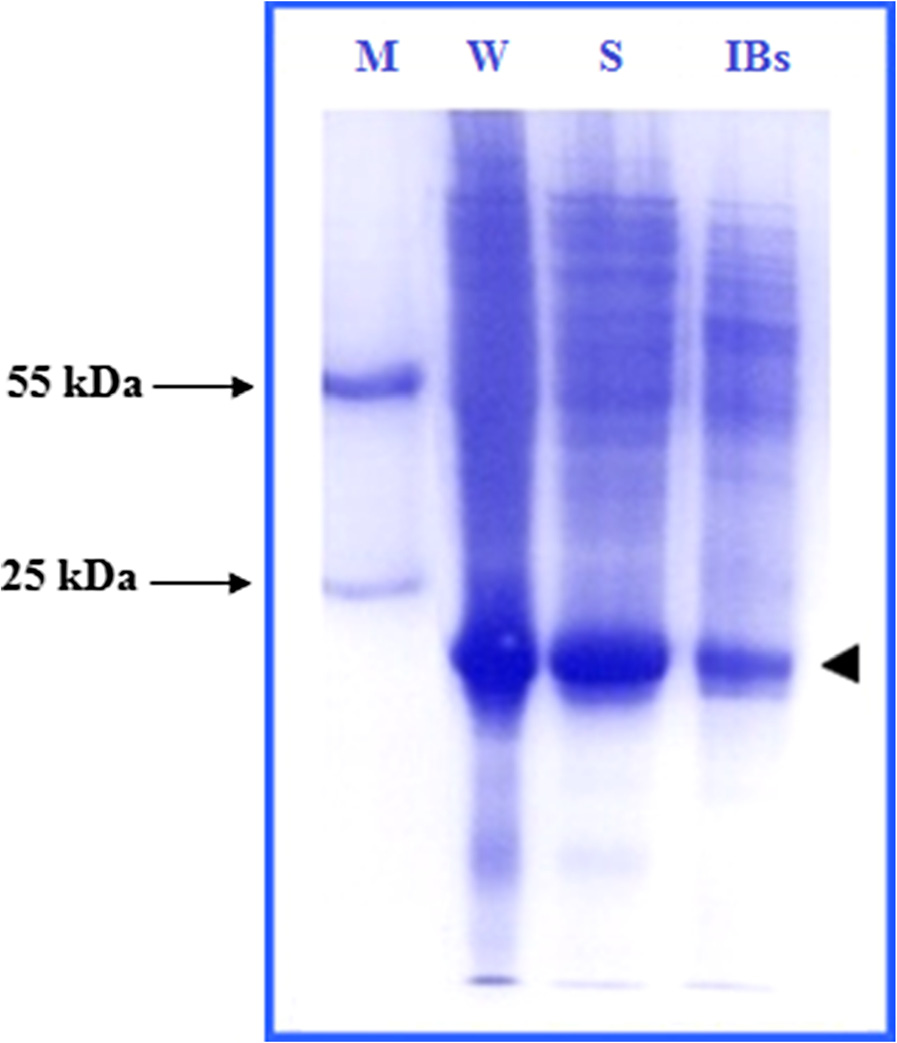
**SDS-PAGE analysis of cIFN-α expressed in the soluble and insoluble inclusion forms.** Lane M: lab-made protein molecular weight marker, W (whole cells), S (soluble fraction of cell lysate), and IBs (inclusion bodies). The arrow head pointed the position of cIFN-α.

Both the supernatant and the pellet of the cell lysate after sonication were examined by SDS–PAGE to detect the solubility of expressed cIFN-α protein. As shown in Figure 5, the main fraction of cIFN-α produced in auto-induction system was found in the soluble form after 24 h of incubation at 25°C or 30°C. Yields of cIFN-α in different cell preparations are reported in Table 2.

**Table 2:** **Yields of cIFN-α expressed in optimized auto-induction system**

**Sample**	**cIFN-α yield (g/L)**
Total cell protein	1.24
Total cell lysate	1.08
Soluble cell lysate	0.76^a^ (70.37%)
Solubilized inclusion bodies	0.217 (20.09%)

^a^Majority of the expressed cIFN-α protein was in the cell lysate supernatant (about 70% of the yield in total cell lysate).

**Figure 6:**
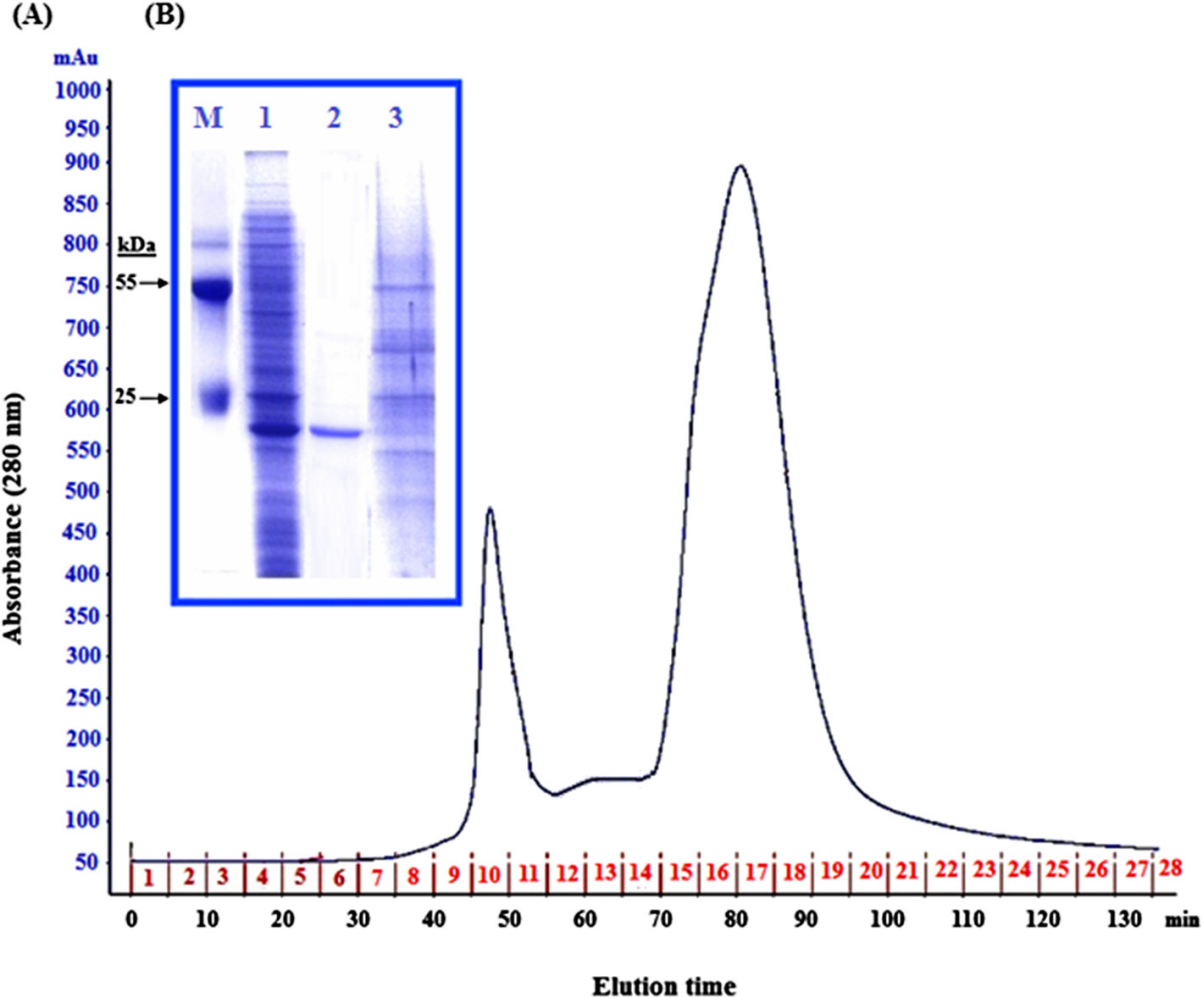
**FPLC purification of recombinant cIFN-α expressed in auto-induction system on a DEAE-Sepharose column. (A)** Protein elution profile (chromatogram). The profile clearly showed three peaks (fractions 8–11, 12–14, and 15–20). Numbers 1–28: number of collected fractions. **(B)** SDS-PAGE analysis of samples before and after cIFN-α purification. Lane M: lab-made protein molecular weight marker. Lane 1: cell lysate supernatant of auto-induced cells after 24 h incubation at 30°C. Lane 2: recovered cIFN-α protein after purification. Lane 3: cell lysate supernatant of uninduced cells.

Our strategy for purifying expressed cIFN-α in auto-induction system involved separation of the soluble fraction of cell lysate and purification of cIFN-α by ion-exchange chromatography. The cell lysate supernatant was loaded onto a DEAE-Sepharose column previously equilibrated with 20 mM Tris–HCl, pH 8. After washing, the bound proteins were eluted with 100 ml linear gradient of 0–1 M NaCl. The elution profile of cIFN-α is shown in Figure 6A. Figure 6B shows SDS-PAGE profile of samples before and after cIFN-α purification. ELISA analysis of samples before (cell lysate supernatant containing cIFN-α) and after cIFN-α purification indicated that there is a significant difference (*P* < 0.05) in ELISA signals between the cell lysate supernatant containing cIFN-α (mean OD at 405 nm ± SD was 0.476 ± 0.023) and the purified cIFN-α (mean OD at 405 nm ± SD was 0.851 ± 0.009). The data revealed that cIFN-α was purified to near homogeneity, utilizing single-step ion-exchange chromatography on DEAE-Sepharose from cell lysate supernatant. The yield was 270 mg of purified cIFN-α from 1 liter of shake flask culture as estimated by Lowry method which is much higher than IPTG-inducible T7 expression system that yielded 150 mg/L (unpublished results) and heat-inducible expression system that achieved a yield of 70 mg/L (as we have previously reported). These results suggest that auto-inducing media achieved 1.8- and 3.8-fold higher recombinant cIFN-α protein yield than IPTG-inducible T7 expression system and heat-inducible expression system, respectively.

### Anticancer activity of cIFN-α expressed by auto-induction

To evaluate the anticancer (antiproliferative) activity of recombinant cIFN-α and commercial IFN-α2a, the inhibitory effect on hepatoma cell line HepG2 was studied using MTT colorimetric assay. The amount of formazan produced by HepG2 cells treated with different concentrations of the tested interferons was compared with the amount of formazan produced by untreated cells, the efficiency of tested interferons in causing death or changing metabolism of cancer cells could be deduced through the significant decrease in the absorbance of purple formazan product at 560 nm after 24 h from treatment with various concentrations of samples.

**Figure 7:**
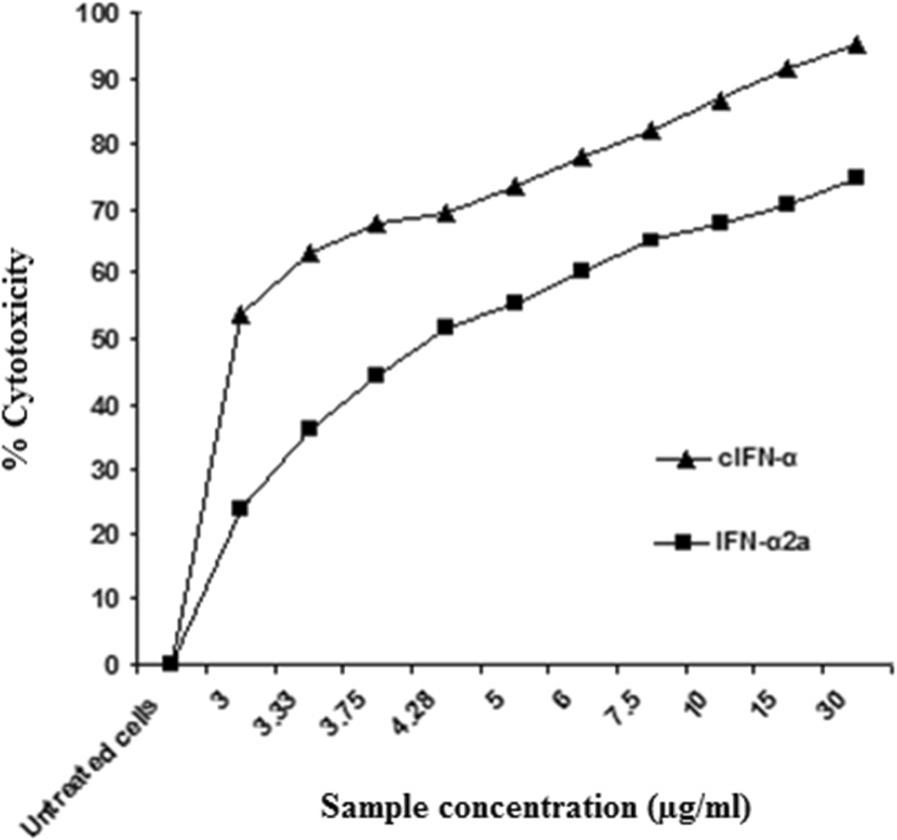
**The inhibitory effect (antiproliferative activity) of cIFN-α synthesized in auto-induction system and commercial IFN-α2a on HepG2 cells.** Both cIFN-α and IFN-α2a have an inhibitory effect on HepG2 cells after 24 h treatment; (LD_50_ = 2.79 μg/ml); (LD_50_ = 4.16 μg/ml), respectively.

The results were expressed as percentage of cytotoxicity compared with the untreated cells. The relationship between percent cytotoxicity and the sample concentration is illustrated in Figure 7. This plot of % cytotoxicity versus sample concentration was used to calculate LD_50_. Recombinant cIFN-α and commercial IFN-α2a demonstrated a potent inhibitory (antiproliferative) effect on HepG2 cell line after 24 h of treatment; (LD_50_ = 2.79 μg/ml and LD_50_ = 4.16 μg/ml, respectively). This was equivalent to 33% (1.5-fold) enhancement in activity. Figure 7 clearly demonstrated the superior anticancer activity of cIFN-α over the commercial IFN-α2a.

### Selectivity index calculation

**Figure 8:**
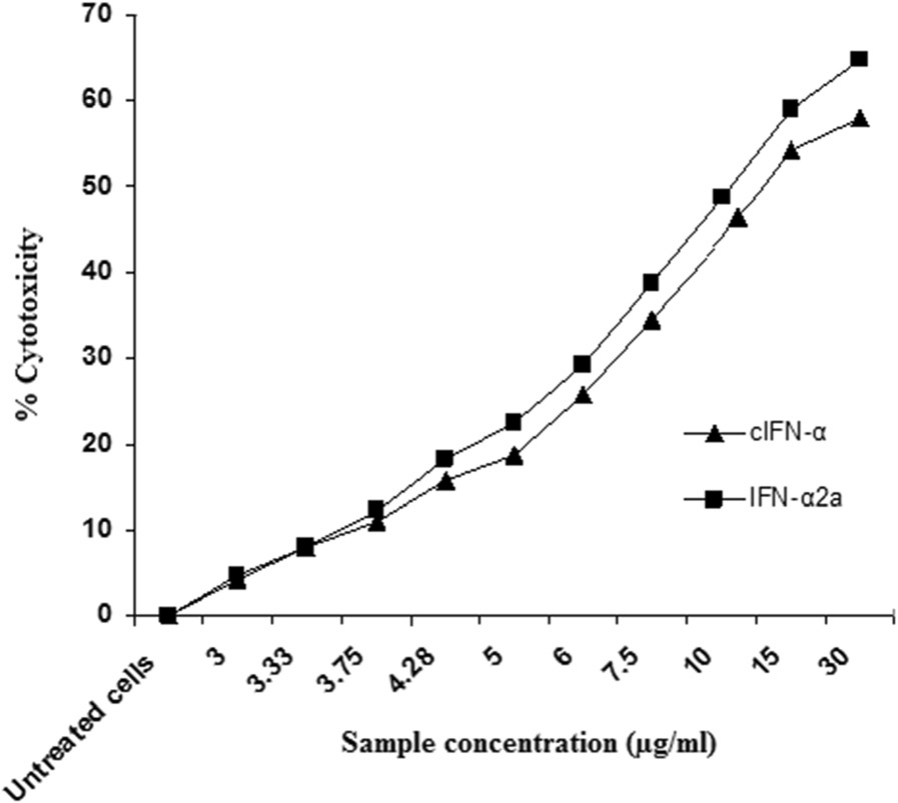
**The inhibitory effect (antiproliferative activity) of cIFN-α synthesized in auto-induction system and commercial IFN-α2a on PBMCs.** The LD_50_ values for cIFN-α and IFN-α2a were 10.63 and 10.16, respectively.

The effect of cIFN-α and IFN-α2a on the proliferation of human lymphocytes was evaluated after 24 h incubation by MTT assay (Figure 8). Both interferons showed significantly lower antiproliferative activity (*P* < 0.05) against PBMCs than that against HepG2 cells. The SI values were calculated using the ratio:

$$ \mathrm{S}\mathrm{I} = {\mathrm{LD}}_{50}\mathrm{of}\ \mathrm{tested}\ \mathrm{s}\mathrm{amples}\ \mathrm{in}\ \mathrm{normal}\ \mathrm{cell}\ \left(\mathrm{lymphocyte}\right)/{\mathrm{LD}}_{50}\mathrm{of}\ \mathrm{tested}\ \mathrm{s}\mathrm{amples}\ \mathrm{in}\ \mathrm{HepG}2\ \mathrm{cell}\mathrm{s}. $$

The SI values for cIFN-α and IFN-α2a were 3.81 and 2.44, respectively. Recombinant cIFN-α showed a significantly higher selectivity (*P* < 0.05) to HepG2 cells than selectivity to HepG2 cells observed in case of commercial IFN-α2a.

### Antiviral activity assay

The antiviral potency of recombinant cIFN-α and commercial IFN-α2a was determined via an *in vitro* assay, by using Vero cells challenged with HSV-1. The results were expressed as EC_50_. The EC_50_ value for recombinant cIFN-α was 30.85 ng/ml. Protection obtained with a total of 30.85 ng/ml of recombinant cIFN-α was equivalent to the protection afforded by 100.35 IU/ml of commercial IFN-α2a. The EC_50_, LD_50_, and therapeutic index values for cIFN-α and IFN-α2a are presented in Table 3. Based on the definition of cIFN-α unit as the ability to protect 50% of cells from HSV-1-induced cytopathogenicity, it was calculated that the final purified cIFN-α has specific antiviral activity of 3.25 × 10^6^ IU/mg protein as determined by cytopathogenicity assay; the units were determined using commercial IFN-α2a.

**Table 3:** **LD**_**50**_ **, EC**_**50**_ **and therapeutic index values of recombinant cIFN-α determined via an** ***in vitro*** **assay, by using Vero cells challenged with HSV-1**

**Interferon**	**LD**_**50**_	**EC**_**50**_	**Therapeutic index (LD**_**50**_ **/EC**_**50**_ **)**
Recombinant cIFN-α	12.68 μg/ml	30.85 ng/ml	411.02
Commercial IFN-α2a	992.81 IU/ml	100.35 IU/ml	9.89

## Discussion

Consensus interferon is a synthetic interferon that reflects most of the human type I interferons. This interferon demonstrates a higher biological capacity *in vitro*. Consensus interferon has been used internationally in treatment of patients with chronic hepatitis C who fail to respond to conventional IFN-α therapy or those who relapse after treatment cessation [30].

We have previously used a heat-inducible, phage λP_L_ promoter-based expression system for high-level expression of recombinant human cIFN-α in shake flask *E. coli* cultures [13]. This system is highly productive, avoids the use of chemical inducers and is easily scalable. However, heat shock and high expression rates resulted in accumulation of the expressed protein into insoluble aggregates in a misfolded and biologically inactive form. Although insoluble misfolded cIFN-α was successfully refolded and purified, *in vitro* refolding yielded reduced amounts of biologically active product (70 mg/L). In another study, we reported synthesis of consensus interferon in cell-free extracts after 2–6 h [19]. Reaction conditions were altered to solve insolubility problems. The cell-free system achieved high-yield expression (400 μg/ml after purification process, which can be scaled up), and remarkably was able to efficiently support the complete maturation of cIFN-α. While cell-free protein synthesis proposes several advantages over cell-based methods, this system is not suitable for large-scale protein production. In the present study, we focused on avoiding the problems associated with the use of IPTG induction or heat induction for high-level expression of cIFN-α through the application of auto-inducing media to achieve higher biologically active product yields.

The results showed that the auto-induction system in which lactose was used as inducer produced higher cIFN-α yield than those with IPTG addition (150 mg/L) or heat induction (70 mg/L). We obtained 270 mg highly purified soluble cIFN-α protein from 1 liter of batch shake flask culture, which is the highest reported yield according to available data with the exception of the yield reported by Babu *et al*. [31] which was 300 mg of purified recombinant human IFN-α obtained from 1 liter of high cell density fed-batch *E. coli* culture. This can be explained by the fact that lactose is not only an inducer but also serves as a source of carbon and energy. In contrast, when IPTG is used as an inducer, the cells experience nutrient starvation, affecting protein expression. In addition, heat induction leads to formation of inclusion bodies that need *in vitro* refolding before purification which increases downstream product loss.

The synthesized cIFN-α in auto-induction system was biologically active as confirmed by its anticancer and antiviral effects. The anticancer effect of cIFN-α exceeds that of the monotype interferon (IFN-α2a), with higher selectivity to the tested cancer cell line, which may agree with the previous results of antiproliferative effect of consensus interferon in comparison to that of natural recombinant alpha interferons [6]. Purified cIFN-α has specific antiviral activity of 3.25 × 10^6^ IU/mg protein.

## Conclusions

In this study, higher product yield was achieved by using the auto-induction process for production of cIFN-α protein in *E. coli*. Auto-induction can be applied to increase the yield of other therapeutic recombinant proteins whose expression in *E. coli* using conventional expression systems is still challenging.

## Abbreviations

BSA: Bovine serum albumin
cIFN-α: Human consensus interferon-alpha
CPE: Cytopathogenicity effect
DAB: 3,3 Diaminobenzidine
DCW: Dry cell weight
DMEM: Dulbecco’s modified Eagle medium
DMSO: Dimethyl sulfoxide
EC_50_: 50% effective concentration
ELISA: Enzyme-linked immunosorbent assay
FBS: Fetal bovine serum
HSV-1: Herpes simplex virus 1
IFNs: Interferons
IPTG: Isopropyl-β-D-1-thiolgalactopyranoside
LB: Luria Bertani
LD_50_: Concentration lethal to 50% of the cells
MTT: 3-(4, 5-dimethylthiazol-2-yl)-2, 5-diphenyltetrazolium bromide
PBMCs: Peripheral blood mononuclear cells
*p*-NPP: *p*-Nitrophenyl phosphate
RBC: Red blood cell
SDS-PAGE: Sodium dodecyl sulfate polyacrylamide gel electrophoresis
SI: Selectivity index
